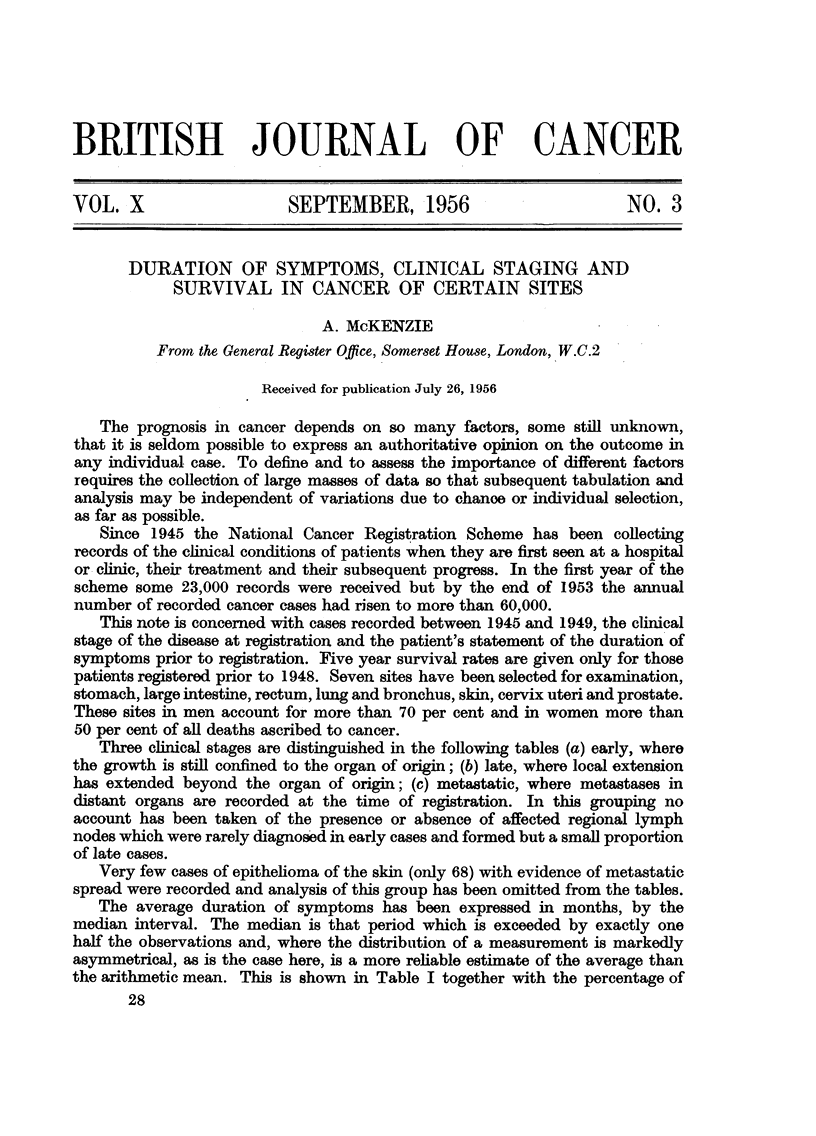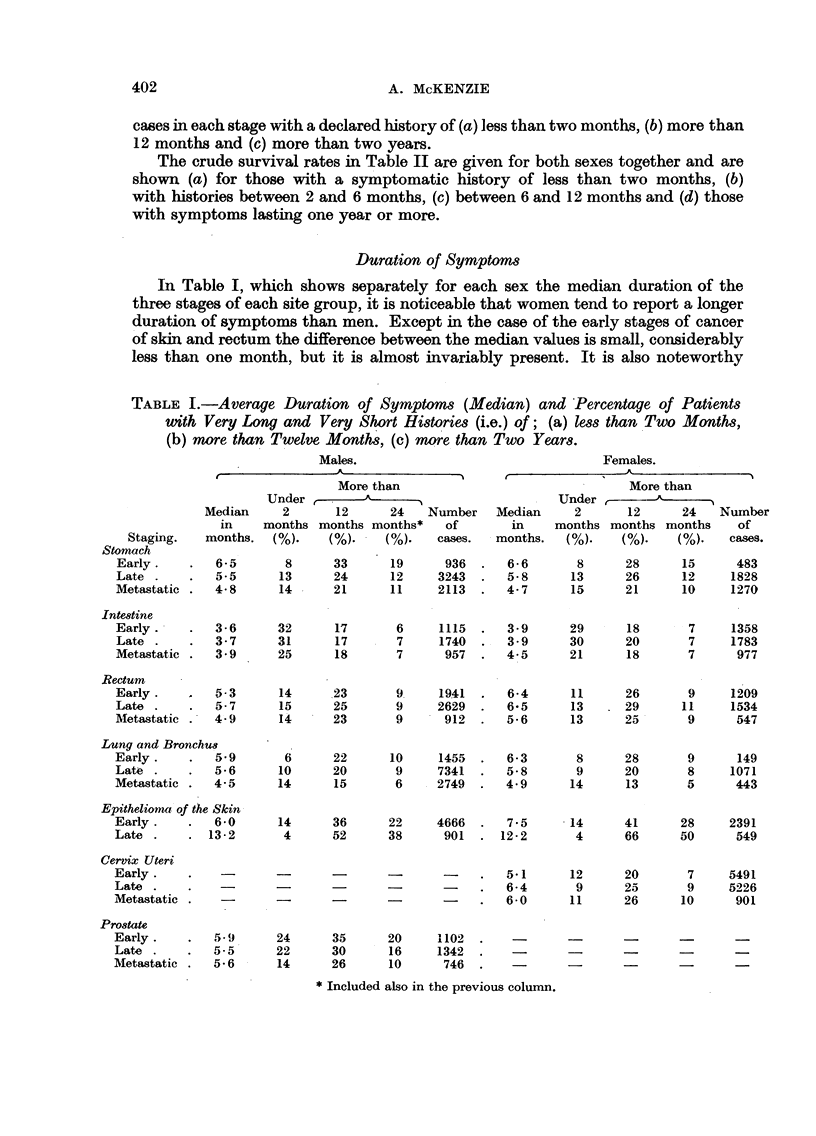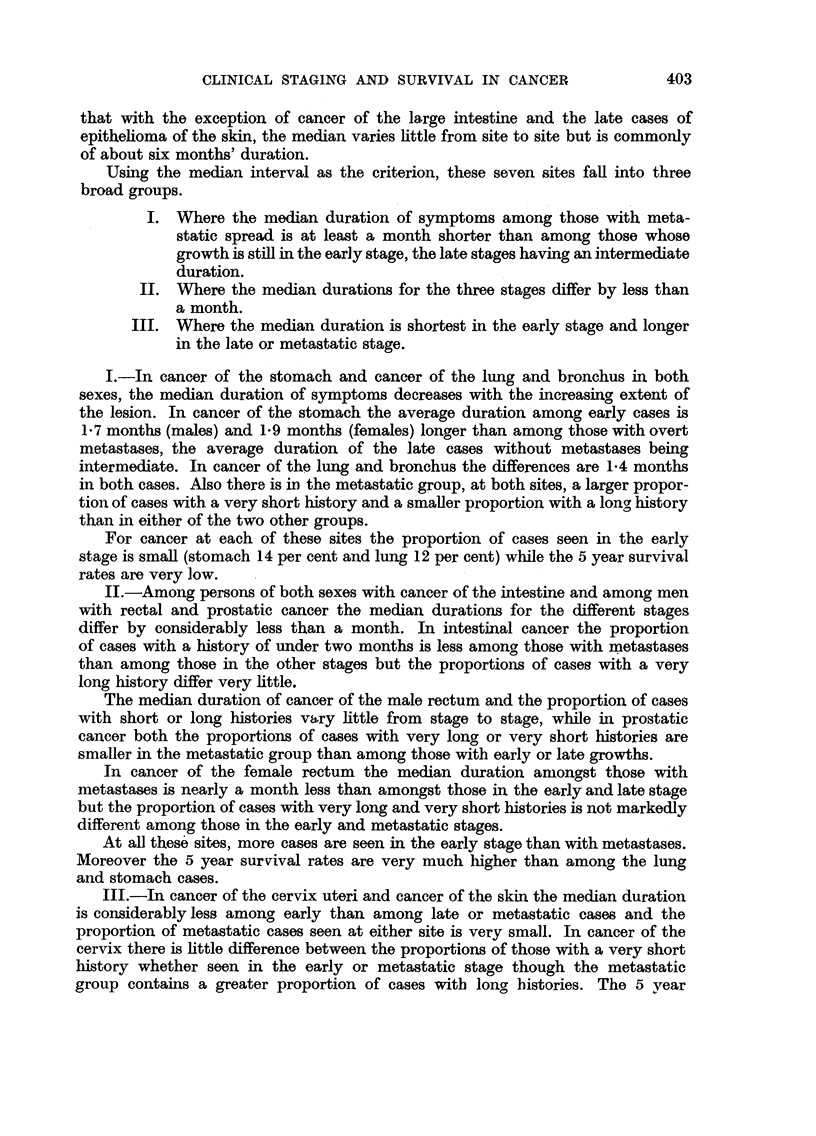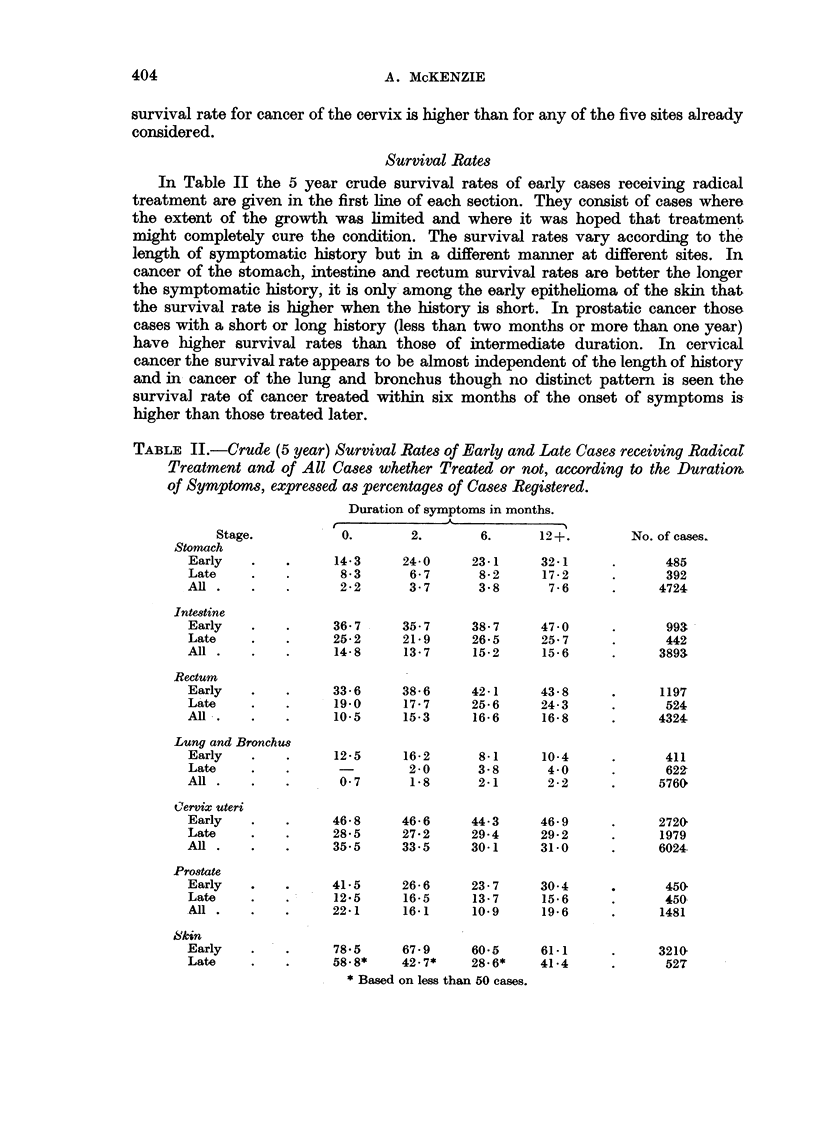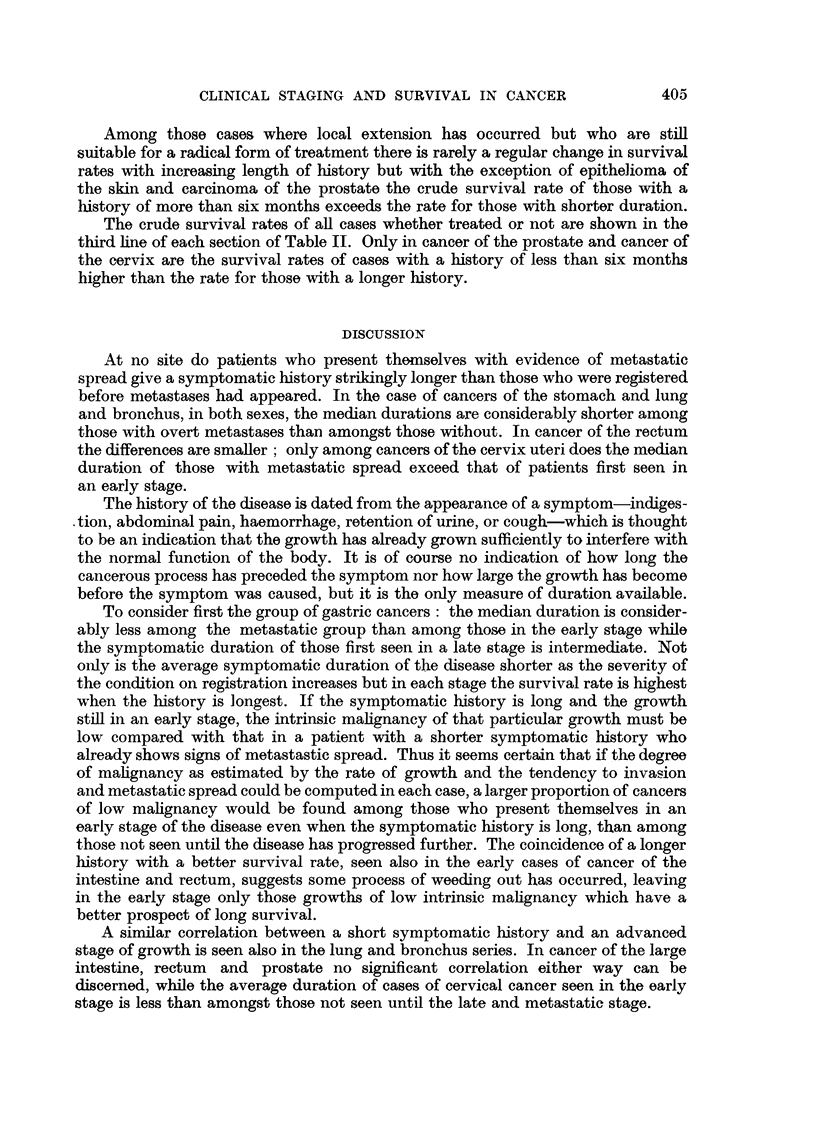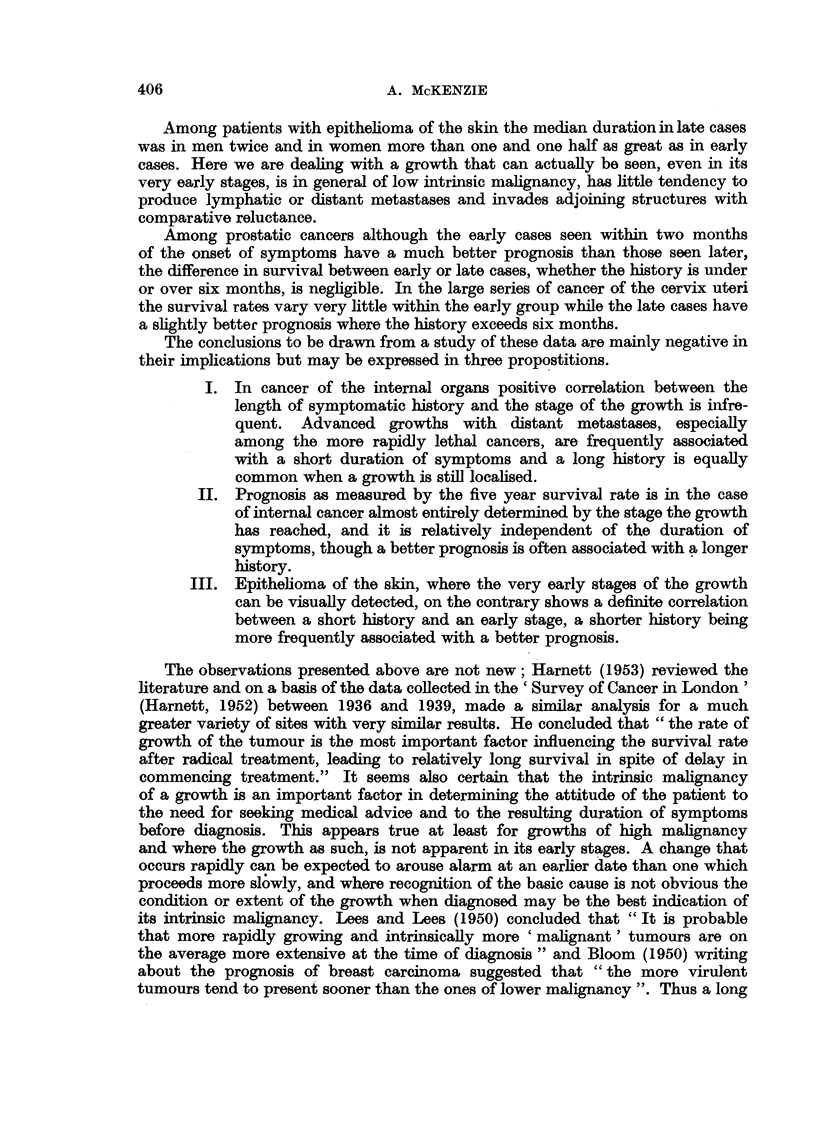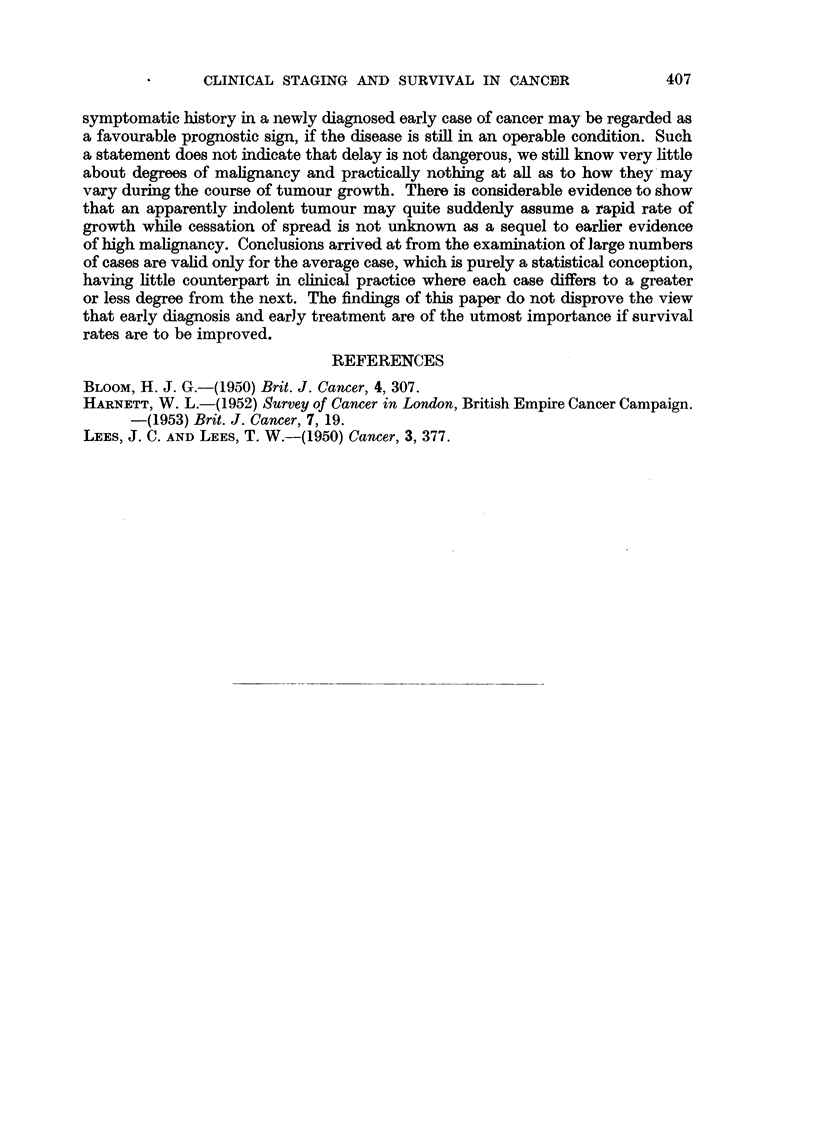# Duration of Symptoms, Clinical Staging and Survival in Cancer of Certain Sites

**DOI:** 10.1038/bjc.1956.46

**Published:** 1956-09

**Authors:** A. McKenzie


BRITISH JOURNAL OF CANCER

VOL. X SEPTEMBER, 1956 NO. 3

### DURATION OF SYMPTOMS, CLINICAL STAGING AND SURVIVAL IN CANCER OF CERTAIN SITES

A. McKENZIE

*From the General Register Office, Somerset House, London, W.C.2*

Received for publication July 26, 1956

The prognosis in cancer depends on so many factors, some still unknown, that it is seldom possible to express an authoritative opinion on the outcome in any individual case. To define and to assess the importance of different factors requires the collection of large masses of data so that subsequent tabulation and analysis may be independent of variations due to chance or individual selection, as far as possible.

Since 1945 the National Cancer Registration Scheme has been collecting records of the clinical conditions of patients when they are first seen at a hospital or clinic, their treatment and their subsequent progress. In the first year of the scheme some 23,000 records were received but by the end of 1953 the annual number of recorded cancer cases had risen to more than 60,000.

This note is concerned with cases recorded between 1945 and 1949, the clinical stage of the disease at registration and the patient's statement of the duration of symptoms prior to registration. Five year survival rates are given only for those patients registered prior to 1948. Seven sites have been selected for examination, stomach, large intestine, rectum, lung and bronchus, skin, cervix uteri and prostate. These sites in men account for more than 70 per cent and in women more than 50 per cent of all deaths ascribed to cancer.

Three clinical stages are distinguished in the following tables (*a*) early, where the growth is still confined to the organ of origin; (*b*) late, where local extension has extended beyond the organ of origin; (*c*) metastatic, where metastases in distant organs are recorded at the time of registration. In this grouping no account has been taken of the presence or absence of affected regional lymph nodes which were rarely diagnosed in early cases and formed but a small proportion of late cases.

Very few cases of epithelioma of the skin (only 68) with evidence of metastatic spread were recorded and analysis of this group has been omitted from the tables.

The average duration of symptoms has been expressed in months, by the median interval. The median is that period which is exceeded by exactly one half the observations and, where the distribution of a measurement is markedly asymmetrical, as is the case here, is a more reliable estimate of the average than the arithmetic mean. This is shown in Table I together with the percentage of cases in each stage with a declared history of (*a*) less than two months, (*b*) more than 12 months and (*c*) more than two years.

The crude survival rates in Table II are given for both sexes together and are shown (*a*) for those with a symptomatic history of less than two months, (*b*) with histories between 2 and 6 months, (*c*) between 6 and 12 months and (*d*) those with symptoms lasting one year or more.

### *Duration of Symptoms*

In Table I, which shows separately for each sex the median duration of the three stages of each site group, it is noticeable that women tend to report a longer duration of symptoms than men. Except in the case of the early stages of cancer of skin and rectum the difference between the median values is small, considerably less than one month, but it is almost invariably present. It is also noteworthy

TABLE I.—*Average Duration of Symptoms (Median) and Percentage of Patients with Very Long and Very Short Histories* (i.e.) *of*; (a) *less than Two Months,* (b) *more than Twelve Months,* (c) *more than Two Years.*

| | Males. | | | | | Females. | | | | |
|---|---|---|---|---|---|---|---|---|---|---|
| | | | More than | | | | | More than | | |
| Staging. | Median in months. | Under 2 months (%). | 12 months (%). | 24 months* (%). | Number of cases. | Median in months. | Under 2 months (%). | 12 months (%). | 24 months (%). | Number of cases. |
| *Stomach* | | | | | | | | | | |
| Early . | 6·5 | 8 | 33 | 19 | 936 | 6·6 | 8 | 28 | 15 | 483 |
| Late . | 5·5 | 13 | 24 | 12 | 3243 | 5·8 | 13 | 26 | 12 | 1828 |
| Metastatic . | 4·8 | 14 | 21 | 11 | 2113 | 4·7 | 15 | 21 | 10 | 1270 |
| *Intestine* | | | | | | | | | | |
| Early . | 3·6 | 32 | 17 | 6 | 1115 | 3·9 | 29 | 18 | 7 | 1358 |
| Late . | 3·7 | 31 | 17 | 7 | 1740 | 3·9 | 30 | 20 | 7 | 1783 |
| Metastatic . | 3·9 | 25 | 18 | 7 | 957 | 4·5 | 21 | 18 | 7 | 977 |
| *Rectum* | | | | | | | | | | |
| Early . | 5·3 | 14 | 23 | 9 | 1941 | 6·4 | 11 | 26 | 9 | 1209 |
| Late . | 5·7 | 15 | 25 | 9 | 2629 | 6·5 | 13 | 29 | 11 | 1534 |
| Metastatic . | 4·9 | 14 | 23 | 9 | 912 | 5·6 | 13 | 25 | 9 | 547 |
| *Lung and Bronchus* | | | | | | | | | | |
| Early . | 5·9 | 6 | 22 | 10 | 1455 | 6·3 | 8 | 28 | 9 | 149 |
| Late . | 5·6 | 10 | 20 | 9 | 7341 | 5·8 | 9 | 20 | 8 | 1071 |
| Metastatic . | 4·5 | 14 | 15 | 6 | 2749 | 4·9 | 14 | 13 | 5 | 443 |
| *Epithelioma of the Skin* | | | | | | | | | | |
| Early . | 6·0 | 14 | 36 | 22 | 4666 | 7·5 | 14 | 41 | 28 | 2391 |
| Late . | 13·2 | 4 | 52 | 38 | 901 | 12·2 | 4 | 66 | 50 | 549 |
| *Cervix Uteri* | | | | | | | | | | |
| Early . | — | — | — | — | — | 5·1 | 12 | 20 | 7 | 5491 |
| Late . | — | — | — | — | — | 6·4 | 9 | 25 | 9 | 5226 |
| Metastatic . | — | — | — | — | — | 6·0 | 11 | 26 | 10 | 901 |
| *Prostate* | | | | | | | | | | |
| Early . | 5·9 | 24 | 35 | 20 | 1102 | — | — | — | — | — |
| Late . | 5·5 | 22 | 30 | 16 | 1342 | — | — | — | — | — |
| Metastatic . | 5·6 | 14 | 26 | 10 | 746 | — | — | — | — | — |

* Included also in the previous column.

that with the exception of cancer of the large intestine and the late cases of epithelioma of the skin, the median varies little from site to site but is commonly of about six months' duration.

Using the median interval as the criterion, these seven sites fall into three broad groups.

I. Where the median duration of symptoms among those with metastatic spread is at least a month shorter than among those whose growth is still in the early stage, the late stages having an intermediate duration.

II. Where the median durations for the three stages differ by less than a month.

III. Where the median duration is shortest in the early stage and longer in the late or metastatic stage.

I.—In cancer of the stomach and cancer of the lung and bronchus in both sexes, the median duration of symptoms decreases with the increasing extent of the lesion. In cancer of the stomach the average duration among early cases is 1·7 months (males) and 1·9 months (females) longer than among those with overt metastases, the average duration of the late cases without metastases being intermediate. In cancer of the lung and bronchus the differences are 1·4 months in both cases. Also there is in the metastatic group, at both sites, a larger proportion of cases with a very short history and a smaller proportion with a long history than in either of the two other groups.

For cancer at each of these sites the proportion of cases seen in the early stage is small (stomach 14 per cent and lung 12 per cent) while the 5 year survival rates are very low.

II.—Among persons of both sexes with cancer of the intestine and among men with rectal and prostatic cancer the median durations for the different stages differ by considerably less than a month. In intestinal cancer the proportion of cases with a history of under two months is less among those with metastases than among those in the other stages but the proportions of cases with a very long history differ very little.

The median duration of cancer of the male rectum and the proportion of cases with short or long histories vary little from stage to stage, while in prostatic cancer both the proportions of cases with very long or very short histories are smaller in the metastatic group than among those with early or late growths.

In cancer of the female rectum the median duration amongst those with metastases is nearly a month less than amongst those in the early and late stage but the proportion of cases with very long and very short histories is not markedly different among those in the early and metastatic stages.

At all these sites, more cases are seen in the early stage than with metastases. Moreover the 5 year survival rates are very much higher than among the lung and stomach cases.

III.—In cancer of the cervix uteri and cancer of the skin the median duration is considerably less among early than among late or metastatic cases and the proportion of metastatic cases seen at either site is very small. In cancer of the cervix there is little difference between the proportions of those with a very short history whether seen in the early or metastatic stage though the metastatic group contains a greater proportion of cases with long histories. The 5 year survival rate for cancer of the cervix is higher than for any of the five sites already considered.

*Survival Rates*

In Table II the 5 year crude survival rates of early cases receiving radical treatment are given in the first line of each section. They consist of cases where the extent of the growth was limited and where it was hoped that treatment might completely cure the condition. The survival rates vary according to the length of symptomatic history but in a different manner at different sites. In cancer of the stomach, intestine and rectum survival rates are better the longer the symptomatic history, it is only among the early epithelioma of the skin that the survival rate is higher when the history is short. In prostatic cancer those cases with a short or long history (less than two months or more than one year) have higher survival rates than those of intermediate duration. In cervical cancer the survival rate appears to be almost independent of the length of history and in cancer of the lung and bronchus though no distinct pattern is seen the survival rate of cancer treated within six months of the onset of symptoms is higher than those treated later.

TABLE II.—*Crude (5 year) Survival Rates of Early and Late Cases receiving Radical Treatment and of All Cases whether Treated or not, according to the Duration of Symptoms, expressed as percentages of Cases Registered.*

| Stage. | Duration of symptoms in months. | | | | No. of cases. |
|---|---|---|---|---|---|
| | 0. | 2. | 6. | 12+. | |
| *Stomach* | | | | | |
| Early | 14·3 | 24·0 | 23·1 | 32·1 | 485 |
| Late | 8·3 | 6·7 | 8·2 | 17·2 | 392 |
| All | 2·2 | 3·7 | 3·8 | 7·6 | 4724 |
| *Intestine* | | | | | |
| Early | 36·7 | 35·7 | 38·7 | 47·0 | 993 |
| Late | 25·2 | 21·9 | 26·5 | 25·7 | 442 |
| All | 14·8 | 13·7 | 15·2 | 15·6 | 3893 |
| *Rectum* | | | | | |
| Early | 33·6 | 38·6 | 42·1 | 43·8 | 1197 |
| Late | 19·0 | 17·7 | 25·6 | 24·3 | 524 |
| All | 10·5 | 15·3 | 16·6 | 16·8 | 4324 |
| *Lung and Bronchus* | | | | | |
| Early | 12·5 | 16·2 | 8·1 | 10·4 | 411 |
| Late | — | 2·0 | 3·8 | 4·0 | 622 |
| All | 0·7 | 1·8 | 2·1 | 2·2 | 5760 |
| *Cervix uteri* | | | | | |
| Early | 46·8 | 46·6 | 44·3 | 46·9 | 2720 |
| Late | 28·5 | 27·2 | 29·4 | 29·2 | 1979 |
| All | 35·5 | 33·5 | 30·1 | 31·0 | 6024 |
| *Prostate* | | | | | |
| Early | 41·5 | 26·6 | 23·7 | 30·4 | 450 |
| Late | 12·5 | 16·5 | 13·7 | 15·6 | 450 |
| All | 22·1 | 16·1 | 10·9 | 19·6 | 1481 |
| *Skin* | | | | | |
| Early | 78·5 | 67·9 | 60·5 | 61·1 | 3210 |
| Late | 58·8* | 42·7* | 28·6* | 41·4 | 527 |

* Based on less than 50 cases.

Among those cases where local extension has occurred but who are still suitable for a radical form of treatment there is rarely a regular change in survival rates with increasing length of history but with the exception of epithelioma of the skin and carcinoma of the prostate the crude survival rate of those with a history of more than six months exceeds the rate for those with shorter duration.

The crude survival rates of all cases whether treated or not are shown in the third line of each section of Table II. Only in cancer of the prostate and cancer of the cervix are the survival rates of cases with a history of less than six months higher than the rate for those with a longer history.

### DISCUSSION

At no site do patients who present themselves with evidence of metastatic spread give a symptomatic history strikingly longer than those who were registered before metastases had appeared. In the case of cancers of the stomach and lung and bronchus, in both sexes, the median durations are considerably shorter among those with overt metastases than amongst those without. In cancer of the rectum the differences are smaller ; only among cancers of the cervix uteri does the median duration of those with metastatic spread exceed that of patients first seen in an early stage.

The history of the disease is dated from the appearance of a symptom—indigestion, abdominal pain, haemorrhage, retention of urine, or cough—which is thought to be an indication that the growth has already grown sufficiently to interfere with the normal function of the body. It is of course no indication of how long the cancerous process has preceded the symptom nor how large the growth has become before the symptom was caused, but it is the only measure of duration available.

To consider first the group of gastric cancers : the median duration is considerably less among the metastatic group than among those in the early stage while the symptomatic duration of those first seen in a late stage is intermediate. Not only is the average symptomatic duration of the disease shorter as the severity of the condition on registration increases but in each stage the survival rate is highest when the history is longest. If the symptomatic history is long and the growth still in an early stage, the intrinsic malignancy of that particular growth must be low compared with that in a patient with a shorter symptomatic history who already shows signs of metastastic spread. Thus it seems certain that if the degree of malignancy as estimated by the rate of growth and the tendency to invasion and metastatic spread could be computed in each case, a larger proportion of cancers of low malignancy would be found among those who present themselves in an early stage of the disease even when the symptomatic history is long, than among those not seen until the disease has progressed further. The coincidence of a longer history with a better survival rate, seen also in the early cases of cancer of the intestine and rectum, suggests some process of weeding out has occurred, leaving in the early stage only those growths of low intrinsic malignancy which have a better prospect of long survival.

A similar correlation between a short symptomatic history and an advanced stage of growth is seen also in the lung and bronchus series. In cancer of the large intestine, rectum and prostate no significant correlation either way can be discerned, while the average duration of cases of cervical cancer seen in the early stage is less than amongst those not seen until the late and metastatic stage.

Among patients with epithelioma of the skin the median duration in late cases was in men twice and in women more than one and one half as great as in early cases. Here we are dealing with a growth that can actually be seen, even in its very early stages, is in general of low intrinsic malignancy, has little tendency to produce lymphatic or distant metastases and invades adjoining structures with comparative reluctance.

Among prostatic cancers although the early cases seen within two months of the onset of symptoms have a much better prognosis than those seen later, the difference in survival between early or late cases, whether the history is under or over six months, is negligible. In the large series of cancer of the cervix uteri the survival rates vary very little within the early group while the late cases have a slightly better prognosis where the history exceeds six months.

The conclusions to be drawn from a study of these data are mainly negative in their implications but may be expressed in three propostitions.

I. In cancer of the internal organs positive correlation between the length of symptomatic history and the stage of the growth is infrequent. Advanced growths with distant metastases, especially among the more rapidly lethal cancers, are frequently associated with a short duration of symptoms and a long history is equally common when a growth is still localised.

II. Prognosis as measured by the five year survival rate is in the case of internal cancer almost entirely determined by the stage the growth has reached, and it is relatively independent of the duration of symptoms, though a better prognosis is often associated with a longer history.

III. Epithelioma of the skin, where the very early stages of the growth can be visually detected, on the contrary shows a definite correlation between a short history and an early stage, a shorter history being more frequently associated with a better prognosis.

The observations presented above are not new ; Harnett (1953) reviewed the literature and on a basis of the data collected in the ' Survey of Cancer in London ' (Harnett, 1952) between 1936 and 1939, made a similar analysis for a much greater variety of sites with very similar results. He concluded that " the rate of growth of the tumour is the most important factor influencing the survival rate after radical treatment, leading to relatively long survival in spite of delay in commencing treatment." It seems also certain that the intrinsic malignancy of a growth is an important factor in determining the attitude of the patient to the need for seeking medical advice and to the resulting duration of symptoms before diagnosis. This appears true at least for growths of high malignancy and where the growth as such, is not apparent in its early stages. A change that occurs rapidly can be expected to arouse alarm at an earlier date than one which proceeds more slowly, and where recognition of the basic cause is not obvious the condition or extent of the growth when diagnosed may be the best indication of its intrinsic malignancy. Lees and Lees (1950) concluded that " It is probable that more rapidly growing and intrinsically more ' malignant ' tumours are on the average more extensive at the time of diagnosis " and Bloom (1950) writing about the prognosis of breast carcinoma suggested that " the more virulent tumours tend to present sooner than the ones of lower malignancy ". Thus a long symptomatic history in a newly diagnosed early case of cancer may be regarded as a favourable prognostic sign, if the disease is still in an operable condition. Such a statement does not indicate that delay is not dangerous, we still know very little about degrees of malignancy and practically nothing at all as to how they may vary during the course of tumour growth. There is considerable evidence to show that an apparently indolent tumour may quite suddenly assume a rapid rate of growth while cessation of spread is not unknown as a sequel to earlier evidence of high malignancy. Conclusions arrived at from the examination of large numbers of cases are valid only for the average case, which is purely a statistical conception, having little counterpart in clinical practice where each case differs to a greater or less degree from the next. The findings of this paper do not disprove the view that early diagnosis and early treatment are of the utmost importance if survival rates are to be improved.

### REFERENCES

BLOOM, H. J. G.—(1950) *Brit. J. Cancer*, **4**, 307.
HARNETT, W. L.—(1952) *Survey of Cancer in London*, British Empire Cancer Campaign.
—(1953) *Brit. J. Cancer*, **7**, 19.
LEES, J. C. AND LEES, T. W.—(1950) *Cancer*, **3**, 377.